# Positron emission tomography PET/CT harmonisation study of different clinical PET/CT scanners using commercially available software

**DOI:** 10.1259/bjro.20190035

**Published:** 2020-06-02

**Authors:** Gerry Lowe, Bruce Spottiswoode, Jerome Declerck, Keith Sullivan, Mhd Saeed Sharif, Wai-Lup Wong, Bal Sanghera

**Affiliations:** 1 Cancer Centre, Mount Vernon Hospital, London, UK; 2 Siemens Medical Solutions USA, Inc., Knoxville, TN, USA; 3 Health Research Methods Unit, University of Hertfordshire, Hertfordshire, UK; 4 School of Arch., Comp. and Eng., University of East London, London, UK; 5 Paul Strickland Scanner Centre, Mount Vernon Hospital, London, UK

## Abstract

**Objectives:**

Harmonisation is the process whereby standardised uptake values from different scanners can be made comparable. This PET/CT pilot study aimed to evaluate the effectiveness of harmonisation of a modern scanner with image reconstruction incorporating resolution recovery (RR) with another vendor older scanner operated in two-dimensional (2D) mode, and for both against a European standard (EARL). The vendor-proprietary software EQ•PET was used, which achieves harmonisation with a Gaussian smoothing. A substudy investigated effect of RR on harmonisation.

**Methods:**

Phantom studies on each scanner were performed to optimise the smoothing parameters required to achieve successful harmonisation. 80 patients were retrospectively selected; half were imaged on each scanner. As proof of principle, a cohort of 10 patients was selected from the modern scanner subjects to study the effects of RR on harmonisation.

**Results:**

Before harmonisation, the modern scanner without RR adhered to EARL specification. Using the phantom data, filters were derived for optimal harmonisation between scanners and with and without RR as applicable, to the EARL standard. The 80-patient cohort did not reveal any statistically significant differences. In the 10-patient cohort SUVmax for RR > no RR irrespective of harmonisation but differences lacked statistical significance (one-way ANOVA F(3.36) = 0.37, *p* = 0.78). Bland-Altman analysis showed that harmonisation reduced the SUVmax ratio between RR and no RR to 1.07 (95% CI 0.96–1.18) with no outliers.

**Conclusions:**

EQ•PET successfully enabled harmonisation between modern and older scanners and against the EARL standard. Harmonisation reduces SUVmax and dependence on the use of RR in the modern scanner.

**Advances in knowledge:**

EQ•PET is feasible to harmonise different PET/CT scanners and reduces the effect of RR on SUVmax.

## Introduction

The recent rate of advance of PET/CT equipment technology has led to the everyday use worldwide of many variants of detector and software.^1,2^ Some earlier PET/CT scanners (*e.g.,* bismuth germanate (BGO) crystal equipment) allow scanning with or without collimation, that is, in 2D or 3D acquisition modes^3^ and these are more prevalent in emerging markets due to healthcare cost factors. However, advanced image reconstruction algorithms including resolution recovery (RR) with point spread function modelling or Bayesian reconstruction^4^ are also in widespread clinical use today.

It is frequently necessary to compare standardised uptake values (SUV) of a lesion between two scans, for example in assessing response to therapy using PERCIST.^5^ Ideally, standardised protocols would be used to ensure comparability between the SUVs; however, it is frequently necessary to compare SUVs acquired in 2D and 3D,^6^ or reconstructed with different algorithms. Harmonisation is the process by which it is assured that SUVs acquired using different scanners or protocols can be compared; if harmonisation is not performed there is a risk of misclassification of lesion response.

SUV variations can arise from factors like patient preparation, scan protocol, data acquisition/processing and image reconstruction/analysis.^7^ Such variations are problematic both in clinical work and in multicentre clinical trials, where multiple imaging systems are used to recruit sufficient patients for statistical power. The process of deriving comparable SUVs from different equipment and protocols is termed harmonisation. Controversy exists in comparing clinical scans acquired with different advanced reconstruction software^8–11^ that has stimulated debate regarding the use of harmonisation schemes.^12^

Attempts to minimise SUV inconsistencies through harmonisation strategies have been recommended by various internationally recognised programmes, for example European Association of Nuclear Medicine (EANM) Research Ltd (EARL) accreditation^13,14^ and the Radiological Society of North America’s (RSNA) North American Quantitative Imaging Biomarkers Alliance (QIBA). Guidelines generally recommend scanning a fillable phantom consisting of hot spheres in a colder background, such as that designed by the Association of Electrical Equipment and Medical Imaging Manufacturers (NEMA) and the International Electrotechnical Commission (IEC). EARL recommends a value and permissible range for the recovery coefficient (RC) for each sphere diameter in the phantom.^15^ Such approaches have proved helpful for achieving harmonisation in clinical trials.

One challenge with such methodology is the potential generation of a separate image set optimised for harmonisation, distinct from the other datasets usually acquired at a centre. This can have consequences for scan labelling, identifying which scan to report, disk storage/data backup and manpower resources. A solution to the challenge of multiple dataset production and associated management has been proposed as the “EQ•PET” proprietary harmonisation software developed by Siemens (*syngo*.MM Oncology, Siemens Medical Solutions USA Inc., Knoxville, TN, United States)^16^.

EQ•PET allows SUV to be harmonised to baseline scans obtained with less spatial resolution (generally on older equipment or software). EQ•PET applies a 3D Gaussian filter to images, using a filter kernel width (“EQ•PET factor”) derived specifically for the particular scanner and protocol in use, that optimally harmonises that scanner and protocol to an agreed standard (*e.g.,* EARL). The EQ•PET factor is usually derived using the NEMA / IEC phantom and is entered directly into the vendor’s software. Smoothing is performed in the background as the original image is displayed, and the harmonised SUV is displayed without additional image creation. Detectability and harmonisation are thus achieved simultaneously. The EQ·PET has been shown to product similar results as EARL,^17^ but without requiring a separate reconstruction.

In this pilot study, we aimed to compare the effects of harmonisation of images from two scanners of different generations: Siemens Biograph-mCT with lutetium oxyorthosilicate (LSO) crystals, including the increased axial field of view (Siemens “TrueV”) which operates without septa in “3D mode” (“scanner A”), and General Electric (GE) (GE Healthcare, Milwaukee, WI, USA) Discovery VCT with BGO crystals (“scanner B”), operated with septa extended between imaging planes in “2D mode”. Harmonisation was performed both between each scanner to the EARL standard, and also between the two scanners, harmonising the newer mCT to the older VCT. A NEMA phantom was used to derive EQ•PET filter parameters. The SUVs measured on clinical images from both scanners were then compared, with and without harmonisation.

## Methods and materials

This study does not constitute clinical research according to the National Health Service England and was therefore conducted as an audit without the need for patient consent or ethical approval.

### Phantom data

The NEMA phantom was prepared without the lung insert as scatter correction was not a prime concern. Spheres and background were filled with F-18 FDG solution concentrations of 45 kBq/ml and 5 kBq/ml respectively, at the time of the first imaging, giving a ratio of 9:1 between these phantom compartments.

The phantom was scanned on both scanners using standard clinical imaging protocols. The scanner A protocol consisted of a 3D PET acquisition, with 3 min/bed, reconstructed using ordered subsets expectation maximisation using time-of-flight information (OSEM + TOF), both with and without RR, with two iterations, 21 subsets, a 200×200 matrix and a Gaussian 5 mm post-filter (referred to hereafter as OSEM + TOF+ RR and OSEM + TOF). The scanner B protocol consisted of a 2D PET acquisition with 4 min/bed, reconstructed using OSEM VuePoint with two iterations, 28 subsets, a 128×128 matrix and a 5.14 mm post-filter. Standard clinical low-dose CT protocols were used for attenuation correction on both scanners. The phantom was scanned six times, three times alternately on each scanner, starting with scanner B. Both scanners had passed manufacturer and nationally approved QA regimes and were serviced at recommended periods.^18^

The following harmonisations were attempted using EQ•PET filtering:

1. To harmonise scanner A OSEM + TOF+ RR data to EARL
2. To harmonise scanner A OSEM + TOF data to EARL
3. To harmonise scanner B data to EARL
4. To harmonise scanner A OSEM + TOF+ RR data to scanner B data

For each harmonisation, an optimised EQ•PET filter parameter was calculated^18^ and entered into the vendor’s dedicated viewing and analysis workstation (*syngo*.MM Oncology, Siemens Medical Solutions USA Inc., Knoxville, TN, United States).

The EARL standard specifies a range in which the RC should reside for each phantom sphere diameter. However, in the case of scanner A harmonisation to scanner B (case four above), no standard upper or lower limits are available to validate the harmonisation success. Therefore for consistency we used the permitted range for each sphere in the EARL recommendations, as a percentage of the RC for that sphere, and applied the same percentage to the scanner B mean RCs to generate a comparable window of acceptance for harmonisation in this case. The phantom-derived EQ•PET filters were then used in the next section with clinical data.

### Clinical data

40 patients with suspected cancer in the head and neck (*n* = 10), lung (*n* = 10), oesophagus (*n* = 5), colorectum (*n* = 8) or lymphatic system (*n* = 7) were consecutively selected from the scanner A database to investigate the influence of different clinical acquisition parameters on the ability of EQ•PET to harmonise suspected lesion SUVmax. Similarly a second independent group of 40 patients with the same number of clinical indications were also consecutively selected from the scanner B database over the same time period. All 80 patients were imaged using routine protocols over a period of approximately 7 months. The tracer used was F-18 FDG throughout. Patients were imaged 90 min after injection.

For scanner A patients, the average ±standard deviation (sd) for weight, age, injected activity and blood glucose was 78.22 kg ±22.98, 64.30 year ±11.66, 347.5 MBq ±97.5 and 6.25 mmol l^−1^ ± 1.17, respectively. For scanner B patients, equivalent values were 77.06 kg ±16.58, 61.74 year ±15.53, 349.58 MBq ±74.84 and 5.96 mmol l^−1^ ± 0.92, respectively. For scanner A, data were acquired at 3 min/bed, and reconstructions were performed into a 200×200 image matrix with two iterations, 21 subsets, TOF and RR, and a Gaussian 5 mm post-filter. For scanner B, data were acquired at 4 min/bed, and reconstructions were performed into a 128×128 image matrix with two iterations, 28 subsets and a 5.14 mm post-filter. These parameters were originally optimised using the NEMA image quality phantom and then checked annually for local use and for use in clinical trials.

A subset of 10 scanner A patients comprising head and neck (*n* = 1), lung (*n* = 5) and lymphoma (*n* = 4) cancer with a range of suspected lesion SUVmax was also arbitrarily selected to investigate the effect of OSEM + TOF+ RR versus OSEM + TOF reconstruction with and without EARL harmonisation.

Radiologists reported PET/CT scans under usual standard-of-care conditions on dedicated vendor-specific workstations (*syngo*.MM Oncology, Siemens Medical Solutions USA Inc., Knoxville, TN, United States or GE, ADW). Lesions were identified by inspection of the images during reporting; clinicians drew VOIs and derived lesion SUVmax accordingly. Comparisons were made of SUVmax pre-harmonisation and post-harmonisation.

SUVmax distributions were evaluated for normality using Shapiro-Wilk test and if necessary log_10_ transformed creating normal distributions allowing one-way analysis of variance (ANOVA) to determine statistical significance between distributions. Because distributions were tested for normality, non-parametric testing was not used in this analysis. Comparison of paired weight, age, injected activity and blood glucose datasets between scanners for non-normal patient demographic data was evaluated using Wilcoxin-signed ranked test. StatsDirect statistical software package (StatsDirect Ltd. StatsDirect statistical software. http://www.statsdirect.com. England: StatsDirect Ltd. 2013) was used for all analysis with a *p*-value of <0.05 considered statistically significant.

## Results

### Phantom data

**Figure 1.**
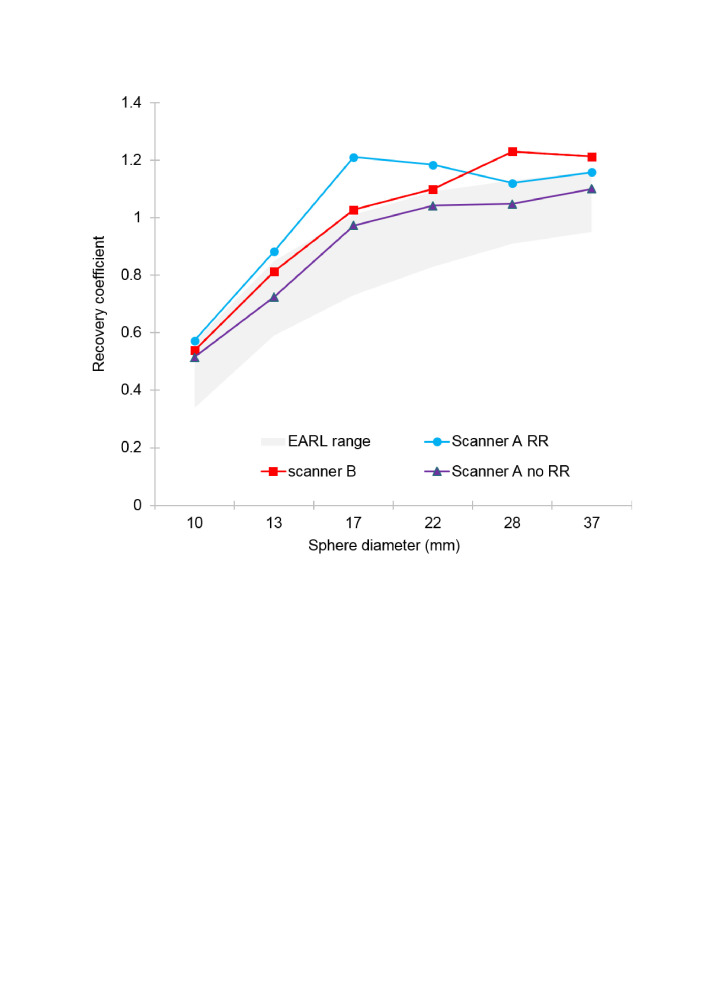
Recovery coefficients before harmonisation compared to European standard criteria

Table 1 shows EQ•PET filter values calculated for optimum harmonisation between scanner B and EARL, scanner A OSEM + TOF+ RR and EARL, scanner A OSEM + TOF and EARL and scanner A OSEM + TOF+ RR to scanner B phantom datasets. Pre-harmonisation phantom RCs for both scanners, with and without RR, compared against the EARL specification are shown in Figure 1. Only the scanner A without RR plot adhered to EARL limits.

**Table 1.** Summary of EQ•PET mean filter values for harmonisation between different data sets

Original dataset	Harmonised to	EQ•PET Filter (mm)
**Scanner B**	EARL	6.0
**Scanner A RR**	EARL	5.7
**Scanner A no RR**	EARL	4.9
**Scanner A RR**	Scanner B	3.6

**Figure 2.**
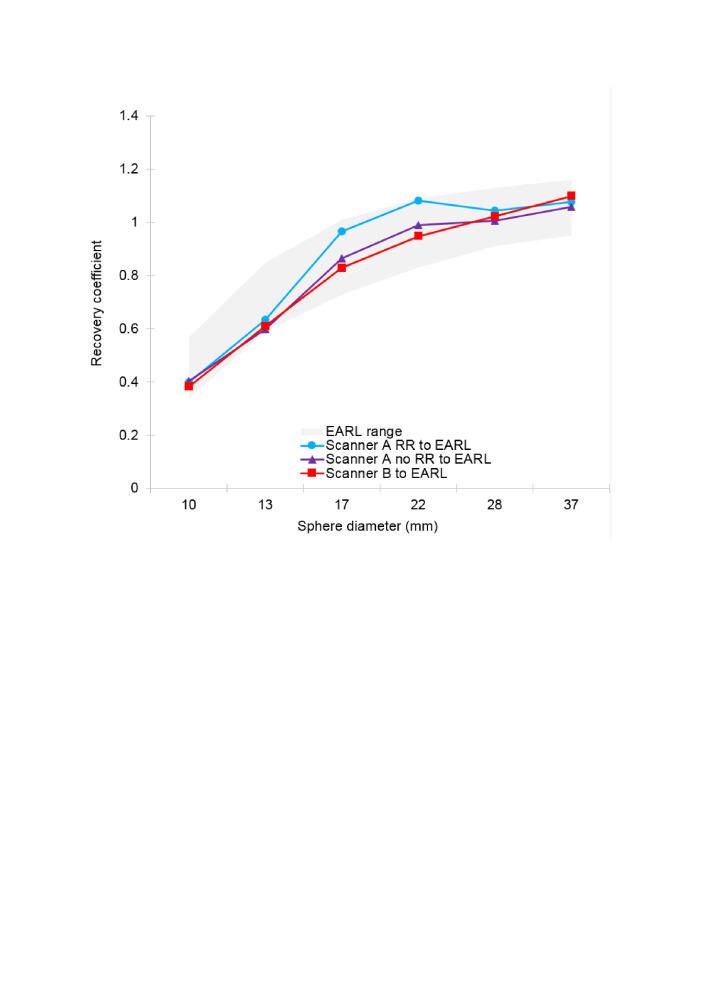
Recovery coefficients after harmonisation compared to European standard criteria

Respective phantom RCs harmonised to the EARL criteria are displayed in Figure 2. All three plots lie within the EARL recommended range, indicating that harmonisation was possible in all cases for all spheres.

**Figure 3.**
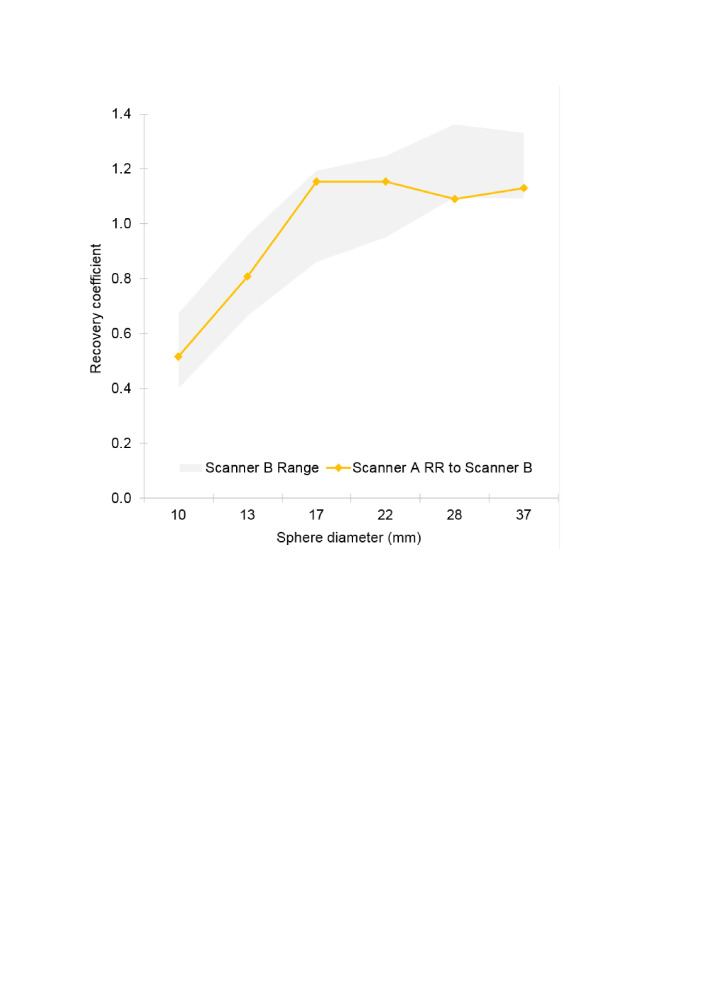
Recovery coefficients after harmonisation with respect to scanner B adapted from European standard criteria

In the case of scanner A harmonised to scanner B (Figure 3), it is seen that scanner A data can be harmonised to lie within a range based on scanner B data, whose width is (arbitrarily) proportionately that of the EARL recommendations. It is noted in the case of the 28 mm sphere the scanner A harmonised RC falls just outside the adopted lower limit by a small percentage difference of 0.58%.

**2. clinical data**

40 different patients were imaged on each scanner. For each scanner, demographic parameters were investigated, some known to influence SUVmax: no significant differences were found between paired patient age (*p* = 0.89), weight (*p* = 0.96), injected activity (*p* = 0.72) or blood glucose (*p* = 0.34). There is therefore a low probability of bias in results presented here arising from variations in patient demographics between scanners.

**Figure 4.**
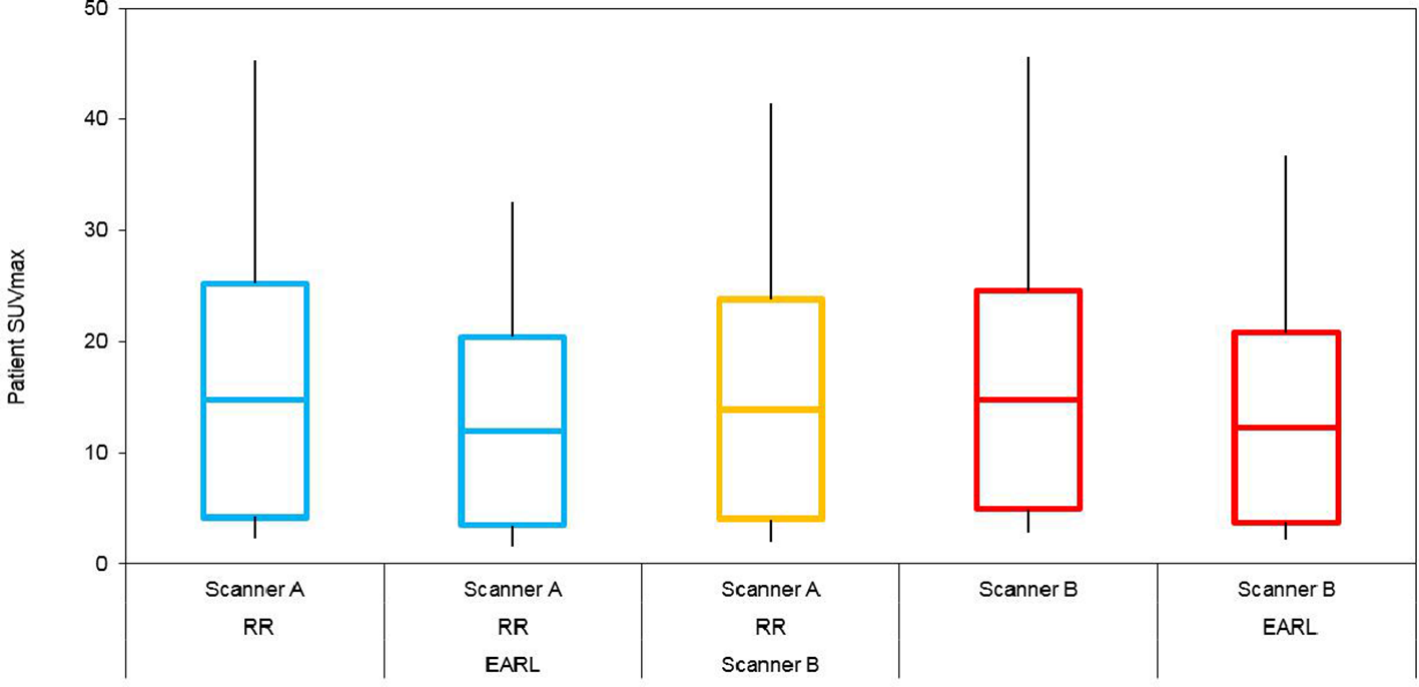
Box and whisker plots depicting min – [mean ± standard deviation] – max from 80 patients showing clinical SUV distributions pre-harmonisation and post-harmonisation

The clinical effect of scanner A RR with or without harmonisation with respect to EARL or scanner B can be seen in the box and whisker plot (Figure 4) depicting min – [mean ± sd] – max clinical SUVmax distributions from 80 suspected oncology patients. This plot also depicts the same information for scanner B with or without harmonisation with respect to EARL specifications. One-way ANOVA revealed no statistically significant differences existed between respective datasets (F(4,195) = 0.82, *p* = 0.51).

**Figure 5.**
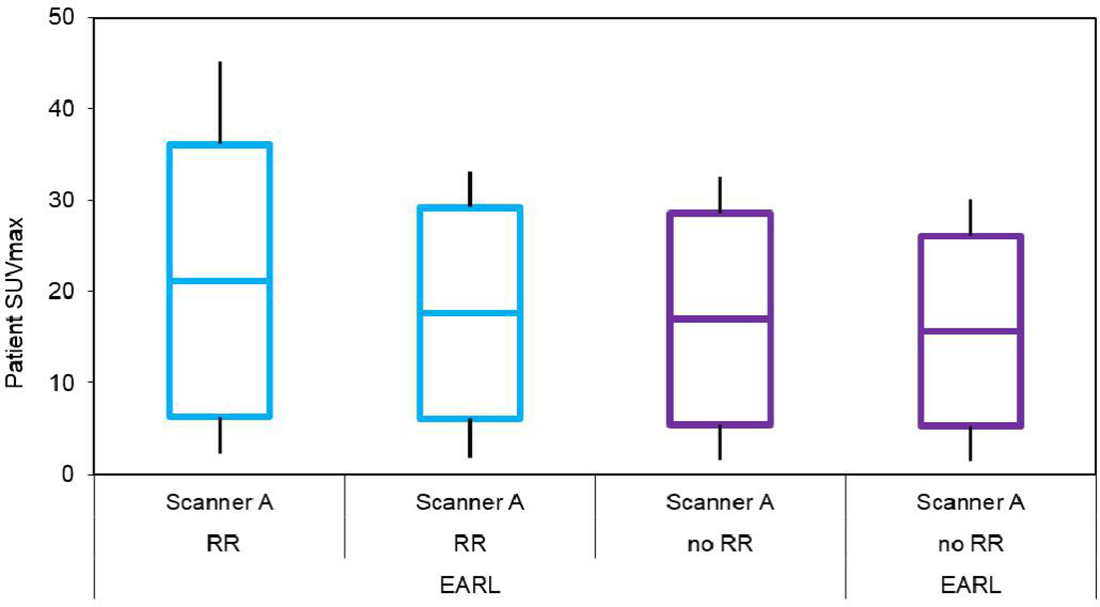
Box and whisker plots depicting min – [mean ± standard deviation] – max from 10 patients showing clinical SUVmax distributions pre-EARL and post-EARL harmonisation with and without RR

Figure 5 compares the effect of scanner A with and without RR with and without harmonisation against EARL criteria for a subset of 10 patients as proof of principle. One-way ANOVA revealed no statistically significant differences between respective datasets (F(3,36) = 0.37, *p* = 0.78).

**Figure 6.**
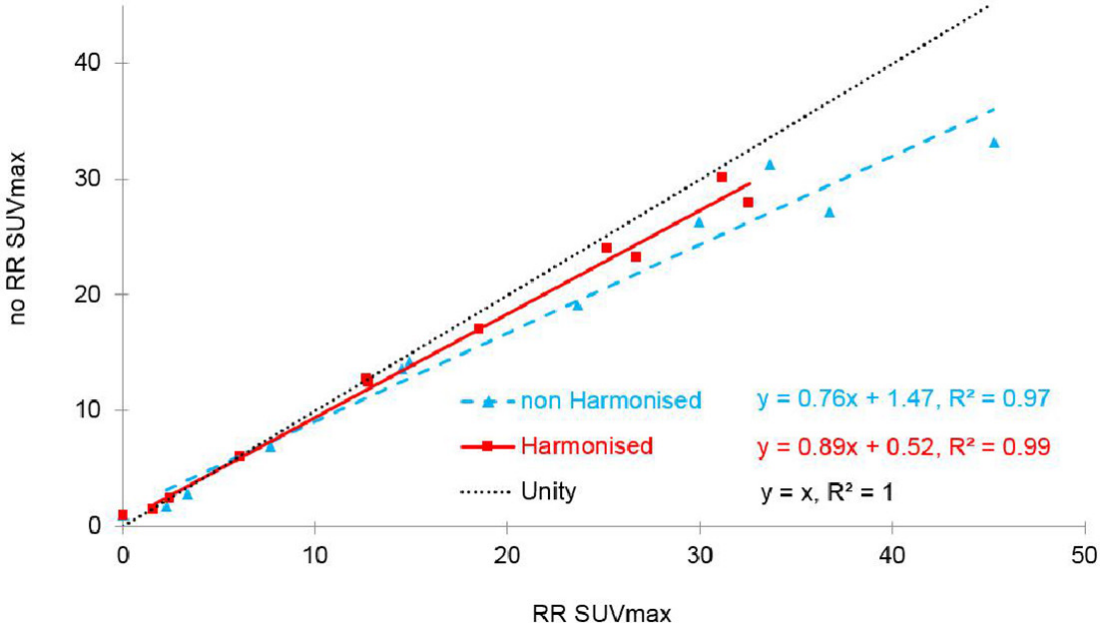
Plot of scanner A no RR SUVmax versus RR SUVmax showing respective unity (black, dotted), with (red, solid) and without (blue, dashed) harmonisation straight line fits plus correlation coefficients for 10 patient cohort.

For this subcohort Figure 6 plots the scanner A data SUVmax with (x-axis) and without (y-axis) RR reconstruction, and both with (square) or without (triangle) harmonisation to the EARL standard for comparison. A good correlation coefficient is achieved both with (r^2^ = 0.99) or without (r^2^ = 0.97) harmonisation. The correlation coefficient must be interpreted bearing in mind that reconstructions with and without RR derive from the same raw data; note although that the level of correlation remains high when harmonisation is applied. The equality line shows that SUVmax with RR is greater than that without RR, irrespective of harmonisation status; however, the slope for harmonised scans (0.89) is greater than that for non-harmonised scans (0.76), indicating that with harmonisation there is less effect on SUVmax when RR is included in the reconstruction.

**Figure 7.**
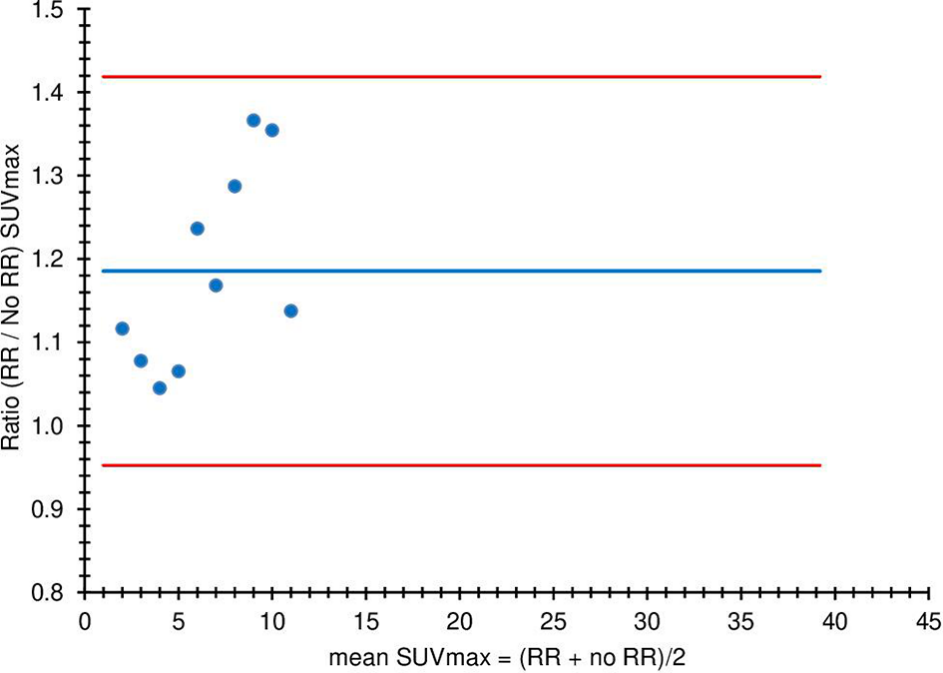
Bland–Altman plot of RR SUVmax versus no RR SUVmax for 10 patients on scanner A without EARL harmonisation

**Figure 8.**
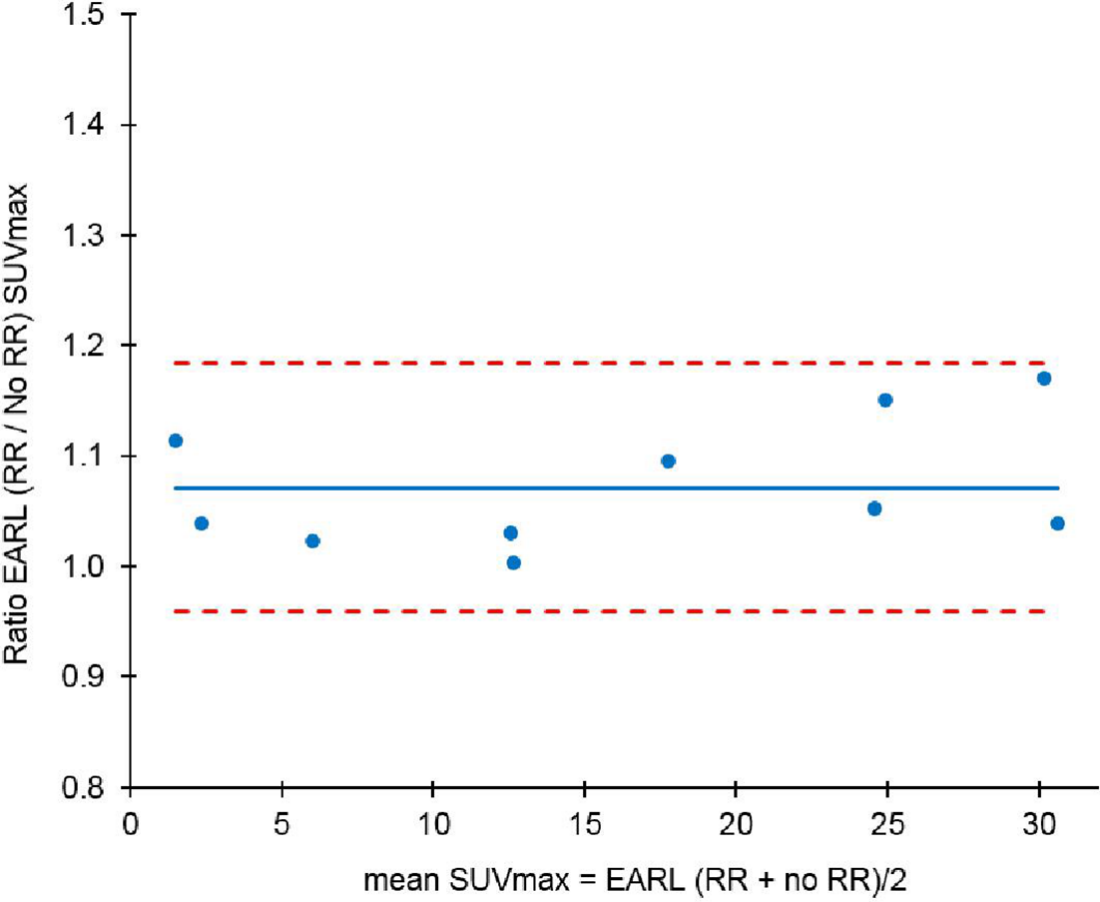
Bland Altman plot of RR SUVmax versus no RR SUVmax for 10 patients on scanner A with EARL harmonisation

Figure 7 depicts the SUVmax relationship between RR and no RR ratio, without EARL harmonisation, using Bland-Altman analysis. The mean ratio was 1.19 (95% CI 0.95–1.42). No outliers were observed beyond the 2xSD CI about the mean. Figure 8 shows the SUVmax relationship between RR and no RR, this time with EARL harmonisation. The mean ratio was 1.07 (95% CI 0.96–1.18). The lower ratio in suggests that harmonisation diminishes the effect of RR on the SUVmax. Again no outliers were observed beyond the 2xSD standard CI about the mean. In both cases, a low (<0.2) Pearson’s correlation coefficient indicates that there is unlikely to be any correlation with SUVmax.

## Discussion

Harmonising scanners in PET/CT centres with different imaging equipment may be of value when scanners have not or cannot be harmonised to recommended guidelines like EARL, but pass all required QA regimes for safe use^19^ and have clinician-approved image quality. During scanner downtime, consistency in patient SUV may be preserved by scanning on different equipment and harmonising SUVs. In addition, harmonisation using modern equipment can play a role immediately following replacement of older technology, where one may wish to ensure consistency of SUV between scanners. EQ•PET harmonisation assists without additional dataset creation in such cases.

We successfully harmonised scanner A data with and without RR to EARL and scanner B data to EARL at a centre that had PET/CT national certification but had not applied for EARL accreditation, Figure 2. In principle, using an acceptable RC range derived from EARL, we also harmonised scanner A RR data to scanner B, although there are some concerns about this expressed in other work.^20^ We found that datasets reconstructed with RR yielded higher mean SUVmax values than datasets reconstructed without RR, but that this difference was less when harmonisation was applied. However, no statistically significant differences in SUVmax were observed between scanner A RR, scanner A RR harmonised to EARL, scanner A RR harmonised to scanner B, scanner B or scanner B harmonised to EARL scanned datasets.

It should be noted that the phantom used for these results did not have the lung insert in place, and this might affect the relationship of 2D to 3D results because the scatter conditions are significantly different between the two modalities. This was not investigated in this work and is a limitation of the study.

SUVmax comparison of clinical datasets from scanner A, with or without RR, and with or without harmonisation was investigated in a subgroup of 10 patients (Figure 5). Mean SUVmax was greater when RR was used, irrespective of harmonisation, but harmonisation to EARL resulted in a lower mean and sd. This again suggests less influence of reconstruction algorithm when harmonisation was applied, although no statistically significant differences were observed between scanner A RR, scanner A RR harmonised to EARL, scanner A without RR or scanner A without RR harmonised to EARL scanned datasets.

The findings in Figure 6 confirm those in Figure 5. RR increases SUVmax in a subset of 10 suspected oncology patients scanned on scanner A, irrespective of harmonisation to EARL, but this effect is reduced by harmonisation.

(Figures 7 and 8) demonstrate the same thing: in the subset of 10 patients, the mean ratio of SUVmax with and without RR is closer to unity (10.6% lower) when harmonisation is used. The confidence limit is also smaller (72.5% lower) with harmonisation.

The EANM/EARL and EQ•PET harmonisation process applied in this pilot study has limitations. Internationally not all PET/CT scanning centres fully conform to EANM-endorsed imaging guidelines but will maintain national QA standards for routine and research scans. Here EARL RCs were based on an average of three scans. Furthermore, RR reconstruction may be differently characterised in simple geometry like the NEMA phantom rather than clinically with variable lesion sizes,^21^ heterogeneous lesions and variable background activity distributions including respiratory motion effects. Other filtering techniques and measurement metrics for example SUVpeak, tumour lesion glycolysis (TLG) or metabolic tumour volume (MTV) may provide additional information in this setting and be less sensitive to reconstruction algorithms,^22^ and it is a specific limitation of the study that only SUVmax is considered. This paper does not consider the frequency with which EQ•PET filter parameters should be checked; however, it is reasonable to assume that this should be done after significant modification to the data acquisition, reconstruction or image processing.^20^

There is growing awareness of PET harmonisation techniques, particularly involving modern technology operated under EANM guidelines against the EARL standard. A recent harmonisation study^23^ used EQ•PET on images from a single scanner, reconstructed using RR and 3D OSEM, of 50 NSCLC patients. Bland-Altman analysis showed that the impact on SUV of the choice of reconstruction algorithm could be reduced without adversely affecting throughput of reconstruction and image interpretation. In another study,^24^ 86 non-small cell lung cancer (NSCLC) patients were imaged for treatment response assessment using three 3D scanners from the same vendor. The authors showed that choice of reconstruction algorithm introduced variability in PERCIST (PET response criteria in solid tumours) response classification, which was minimised by the use of harmonisation to EARL with EQ•PET using lean body mass-based SUVpeak.

In a further NSCLC study^25^ describing different generation scanners, different reconstruction parameters were used to confirm successful EARL harmonisation via Bland-Altman analysis. In a different study,^26^ 517 oncology patients with NSCLC, non-Hodgkin lymphoma and metastatic colorectal cancer were scanned on three different 3D scanner models but from the same manufacturer. Bland-Altman results showed EARL harmonisation with EQ•PET was successful for RR, TOF and OSEM algorithms using SUVmax, SUVmean and SUVpeak.

Another review^27^ endorsed PET/CT EARL harmonisation methodology using EQ•PET in the oncology therapy setting whilst validating accreditation methodology in 51 participating centres. In a new publication,^20^ four modern TOF-enabled and RR-enabled 3D PET/CT scanners from three different vendors were used to demonstrate that harmonisation of phantom studies to EARL specifications could be achieved. However, different RCs were advocated and a modification to multicentre accreditation was recommended as a consequence of advanced reconstruction algorithms.

A recent forward-facing review^28^ investigated adapting harmonisation to other radiopharmaceuticals for example Ga-68-PSMA in prostate cancer for lymph node detection. Literature also exists relating to advanced image analysis techniques like textural analysis^29^ with PET/CT^30^ which require consistency between SUV acquired under different conditions. In a single 3D scanner study^31^ with 71 lesions in 60 NSCLC patients comparable SUV and heterogeneity information was achieved using RR and harmonising with Gaussian smoothing, and then compared to simpler OSEM reconstructed scans.

The majority of clinical studies using EQ•PET have been based on NSCLC, with Siemens modern 3D scanners using LSO-based crystal detector technology. There is little reported about harmonisation between truly different generations of PET/CT scanner, especially across different vendors.

The retrospective cohort of 80 patients in this study demonstrated the clinical feasibility of harmonisation between two scanners and against the EARL standard. Future studies are suggested with larger patient numbers combined with varied scanners from different vendors for further analysis of the clinical consequences of harmonisation. We encourage the adoption of the EQ•PET concept to promote harmonisation without creation of multiple datasets.

## Conclusion

As a PET/CT centre approved for national research studies, we have shown in a pilot study that different PET/CT scanners can be harmonised using EQ•PET to current EARL specifications. Our results are in accordance with others. No statistically significant differences among 80 suspected oncology patient lesion SUVmax with or without harmonisation between scanners was observed. Standard clinical protocols with or without RR reconstruction had lower SUVmax mean and sd following respective harmonisation. A subset of 10 patients reconstructed with or without RR and with or without harmonisation revealed smaller mean and sd in harmonised scans, but again differences were not statistically significant. Harmonisation with EQ•PET is shown to reduce dependence on reconstruction algorithm in both older and modern scanners.

## References

[1] European Society of radiology (ESR). renewal of radiological equipment. Insights into Imaging 2014; 5: 543–6. 25230589 10.1007/s13244-014-0345-1 PMC4195838

[2] Pavlek IB, Brnic Z, Schmidt S, Krpan T, Kralik I Old and outdated radiology equipment in Croatia-radiation safety and economic consequences. Insights Imaging 2016; 7: 283–4. doi: 10.1007/s13244-016-0464-y 26883136 PMC4805625

[3] Graham MM, Badawi RD, Wahl RL Variations in PET/CT methodology for oncologic imaging at U.S. academic medical centers: an imaging response assessment team survey. J Nucl Med 2011; 52: 311–7. doi: 10.2967/jnumed.109.074104 21233185 PMC3889016

[4] Yamaguchi S, Wagatsuma K, Miwa K, Ishii K, Inoue K, Fukushi M Bayesian penalized-likelihood reconstruction algorithm suppresses edge artifacts in PET reconstruction based on point-spread-function. Phys Med 2018; 47: 73–9. doi: 10.1016/j.ejmp.2018.02.013 29609821

[5] Wahl RL, Jacene H, Kasamon Y, Lodge MA From RECIST to PERCIST: evolving considerations for PET response criteria in solid tumors. J Nucl Med 2009; 50 Suppl 1(Suppl 1): 122S–50. doi: 10.2967/jnumed.108.057307 19403881 PMC2755245

[6] Nogueira SA, Dimenstein R, Cunha ML, Wagner J, Funari MBG, Lederman HM Low-Dose radiation protocol using 3D mode in a BGO PET/CT. Radiol Med 2015; 120: 251–5. doi: 10.1007/s11547-014-0422-z 24903708

[7] Keyes JW SUV: standard uptake or silly useless value? J Nucl Med 1995; 36: 1836–9. 7562051

[8] Barrington SF, Sulkin T, Forbes A, Johnson PWM All that glitters is not gold - new reconstruction methods using Deauville criteria for patient reporting. Eur J Nucl Med Mol Imaging 2018; 45: 316–7. doi: 10.1007/s00259-017-3893-z 29198033

[9] Lasnon C, Enilorac B, Aide N Reply to: "All that glitters is not gold - new reconstruction methods using Deauville criteria for patient reporting". Eur J Nucl Med Mol Imaging 2018; 45: 878–81. doi: 10.1007/s00259-018-3938-y 29473107

[10] Enilorac B, Lasnon C, Nganoa C, Fruchart C, Gac A-C, Damaj G, et al Does PET reconstruction method affect Deauville score in lymphoma patients? J Nucl Med 2018; 59: 1049–55. doi: 10.2967/jnumed.117.202721 29242403

[11] Boellaard R, Kobe C, Zijlstra JM, Mikhaeel NG, Johnson PWM, Müller S, et al Does PET reconstruction method affect Deauville scoring in lymphoma patients? J Nucl Med 2018; 59: 1167–9. doi: 10.2967/jnumed.118.211607 29626118

[12] Ferretti A, Chondrogiannis S, Rampin L, Bellan E, Marzola MC, Grassetto G, et al How to harmonize SUVs obtained by hybrid PET/CT scanners with and without point spread function correction. Phys Med Biol 2018; 63: 235010. doi: 10.1088/1361-6560/aaee27 30474620

[13] Boellaard R, Delgado-Bolton R, Oyen WJ, Giammarile F, Tatsch K, Eschner W, et al European association of nuclear medicine (EANM). FDG PET/CT: EANM procedure guidelines for tumour imaging: version 2.0. Eur J Nucl Med Mol Imaging 201: 328–54. 10.1007/s00259-014-2961-x PMC4315529 25452219

[14] Ltd ER EARL): FDG-PET/CT accreditation.. accessed March 2020.

[15] EARL accreditation specifications.. referenced on 24-July-19.

[16] Kelly MD, Declerck JM SUVref: reducing reconstruction-dependent variation in PET SUV. EJNMMI Res 2011; 1: 16. doi: 10.1186/2191-219X-1-16 22214348 PMC3251007

[17] Lasnon C, Salomon T, Desmonts C, Dô P, Oulkhouir Y, Madelaine J, et al Generating harmonized SUV within the EANM EARL accreditation program: software approach versus EARL-compliant reconstruction. Ann Nucl Med 2017; 31: 125–34. doi: 10.1007/s12149-016-1135-2 27812791

[18] EQ•PET: Achieving NEMA - referenced SUV Across Technologies.. Available from: https : // usa.healthcare.siemens.com / molecular-imaging/eq-pet-thank-you referenced on 24-July-19.

[19] Institute of physics and engineering and medicine (IPEM) report no. 108 Quality Assurance of PET and PET/CT Systems.

[20] Kaalep A, Sera T, Rijnsdorp S, Yaqub M, Talsma A, Lodge MA, et al Feasibility of state of the art PET/CT systems performance harmonisation. Eur J Nucl Med Mol Imaging 2018; 45: 1344–61. doi: 10.1007/s00259-018-3977-4 29500480 PMC5993859

[21] Munk OL, Tolbod LP, Hansen SB, Bogsrud TV Point-spread function reconstructed PET images of sub-centimeter lesions are not quantitative. EJNMMI Phys 2017; 4: 5. doi: 10.1186/s40658-016-0169-9 28091957 PMC5236043

[22] Armstrong IS, Kelly MD, Williams HA, Matthews JC Impact of point spread function modelling and time of flight on FDG uptake measurements in lung lesions using alternative filtering strategies. EJNMMI Phys 2014; 1: 99. doi: 10.1186/s40658-014-0099-3 26501457 PMC4545221

[23] Lasnon C, Salomon T, Desmonts C, Dô P, Oulkhouir Y, Madelaine J, et al Generating harmonized SUV within the EANM EARL accreditation program: software approach versus EARL-compliant reconstruction. Ann Nucl Med 2017; 31: 125–34. doi: 10.1007/s12149-016-1135-2 27812791

[24] Quak E, Le Roux P-Y, Lasnon C, Robin P, Hofman MS, Bourhis D, et al Does PET SUV harmonization affect PERCIST response classification? J Nucl Med 2016; 57: 1699–706. doi: 10.2967/jnumed.115.171983 27283930

[25] Lasnon C, Desmonts C, Quak E, Gervais R, Do P, Dubos-Arvis C, et al Harmonizing SUVs in multicentre trials when using different generation PET systems: prospective validation in non-small cell lung cancer patients. Eur J Nucl Med Mol Imaging 2013; 40: 985–96. doi: 10.1007/s00259-013-2391-1 23564036 PMC3679414

[26] Quak E, Le Roux P-Y, Hofman MS, Robin P, Bourhis D, Callahan J, et al Harmonizing FDG PET quantification while maintaining optimal lesion detection: prospective multicentre validation in 517 oncology patients. Eur J Nucl Med Mol Imaging 2015; 42: 2072–82. doi: 10.1007/s00259-015-3128-0 26219870 PMC4623085

[27] Aide N, Lasnon C, Veit-Haibach P, Sera T, Sattler B, Boellaard R EANM/EARL harmonization strategies in PET quantification: from daily practice to multicentre oncological studies. Eur J Nucl Med Mol Imaging 2017; 44(Suppl 1): 17–31. doi: 10.1007/s00259-017-3740-2 PMC5541084 28623376

[28] van der Vos CS, Koopman D, Rijnsdorp S, Arends AJ, Boellaard R, van Dalen JA, et al Quantification, improvement, and harmonization of small lesion detection with state-of-the-art PET. Eur J Nucl Med Mol Imaging 2017; 44(Suppl 1): 4–16. doi: 10.1007/s00259-017-3727-z 28687866 PMC5541089

[29] Davnall F, Yip CSP, Ljungqvist G, Selmi M, Ng F, Sanghera B, et al Assessment of tumor heterogeneity: an emerging imaging tool for clinical practice? Insights Imaging 2012; 3: 573–89. doi: 10.1007/s13244-012-0196-6 23093486 PMC3505569

[30] Hatt M, Tixier F, Pierce L, Kinahan PE, Le Rest CC, Visvikis D Characterization of PET/CT images using texture analysis: the past, the present… any future? Eur J Nucl Med Mol Imaging 2017; 44: 151–65. doi: 10.1007/s00259-016-3427-0 27271051 PMC5283691

[31] Lasnon C, Majdoub M, Lavigne B, Do P, Madelaine J, Visvikis D, et al ^18^F-FDG PET/CT heterogeneity quantification through textural features in the era of harmonisation programs: a focus on lung cancer. Eur J Nucl Med Mol Imaging 2016; 43: 2324–35. doi: 10.1007/s00259-016-3441-2 27325312

